# Tracking the 2022 Hunga Tonga‐Hunga Ha'apai Aerosol Cloud in the Upper and Middle Stratosphere Using Space‐Based Observations

**DOI:** 10.1029/2022GL100091

**Published:** 2022-10-04

**Authors:** G. Taha, R. Loughman, P. R. Colarco, T. Zhu, L. W. Thomason, G. Jaross

**Affiliations:** ^1^ Morgan State University Baltimore MD USA; ^2^ NASA Goddard Space Flight Center Greenbelt MD USA; ^3^ Hampton University Hampton VA USA; ^4^ Science Systems and Applications, Inc. Lanham MD USA; ^5^ NASA Langley Research Center Hampton VA USA

**Keywords:** OMPS LP, aerosols, stratosphere, volcanic eruption, SAGE III/ISS, hunga Tonga

## Abstract

On 15 January 2022, the submarine Hunga Tonga volcanic eruption lofted materials high into the upper stratosphere, reaching a record‐breaking altitude of ∼58 km, unprecedented in the satellite observations era. Within two weeks, the bulk of the injected material circulated the globe between 20–30 km altitude, as observed by satellite instruments. We estimate that the stratospheric aerosol optical depth (sAOD) is the largest since the Pinatubo eruption and is at least twice as great as the sAOD after the 2015 Calbubo eruption despite the similar SO_2_ injection from that eruption. We use space‐based observations to monitor the Hunga‐Tonga volcanic plume evolution and transport at different altitudes as it circulates the globe. While the main aerosol layer remains trapped in the tropical pipe, small parts have already made it to both the northern and southern hemisphere poles by April, which is almost certain to influence this year's ozone hole.

## Key Points

- Ozone Monitoring and Profiler Suite measured the Hunga Tonga aerosol layer in the upper stratosphere at altitudes up to 50 km, and tracked it above 36 km for 2.5 months
- This eruption resulted in the highest stratospheric aerosol optical depth since Pinatubo in 1991
- Within weeks, parts of the volcanic cloud reached the Southern Hemisphere pole, and by April, it also reached the Northern Hemisphere pole

## 1 Introduction

In January 2022, the submarine volcano Hunga Tonga‐Hunga Ha'apai (20.55°S, 175.4°W) erupted twice, sending material high into the stratosphere. The first volcanic plume on 13 January reached an altitude between 18 and 20 km. On 15 January, a second and more powerful series of explosions started at 4:10 UTC and lasted 11 hr, generating airborne shockwaves and oceanic tsunami waves that traveled around the globe (https://www.nesdis.noaa.gov/news/the-hunga-tonga-hunga-haapai-eruption-multi-hazard-event). The eruption lofted material high in the upper stratosphere, reaching an altitude of 55–58 km (Carr et al., 2022; Proud et al., 2022), the highest observed by space‐based measurements, creating an umbrella cloud with radius ∼ 500 km. Until this year, the 1991 eruption of Mount Pinatubo, Philippines, had the highest altitude volcanic injection recorded in the satellite era, which reached 40 km (Holasek et al., 1996). It is unlikely that this eruption will have significant aerosol‐driven climate effects because of the relatively low SO_2_ injection, 400,000 tonnes compared to 20 million tonnes for Pinatubo (Witze, 2022). Millán et al. (2022) estimated that this eruption injected 146 Tg (1 Tg = 1 million tonnes) of water into the stratosphere and predicted that it would result in surface warming rather than surface cooling expected from the sulfate aerosol alone. Thus, because of the extraordinary nature of the eruption, it is essential that we monitor the initial impact and transport of the volcanic plume as it circulates the globe to understand the long‐term effect of this eruption. We expect it to influence Earth's radiative balance and affect the chemical and dynamical processes related to ozone destruction in the stratosphere.

This paper focuses on observations by NASA's OMPS LP instrument, which has previously made observations of smaller eruptions including Kelut (2014), Calbuco (2015), Ambae (2018), Ulawun (2019) and Raikoke (2019) (Gorkavyi et al., 2021; Kloss et al., 2020, 2021; Tadiga et al., 2022). Due to the unusual strength of the eruption, aerosol is observed at altitudes that far exceed the normalization altitude for the standard OMPS LP aerosol retrieval algorithm (above which aerosol scattering is assumed to be negligible). A modified version of the standard processing algorithm was therefore required. Section 2 provides a brief description of the OMPS LP instrument and the modification made to the algorithm. Section 3 describes OMPS LP observations of the volcanic cloud, both during the early days after the main eruption and over its evolution during the next 4 months. It also investigates the volcanic aerosol layer optical properties using various satellite measurements. Section 4 provides a summary and conclusion.

## 2 Methods

### 2.1 Instrument and Algorithm Descriptions

The Ozone Mapping and Profiler Suite (OMPS), which was launched in October 2011 onboard the Suomi National Polar‐orbiting Partnership (S‐NPP) satellite (Flynn et al., 2006), consists of three instruments designed to measure the ozone layer. One of the three instruments, the limb profiler (LP), can provide relatively high‐vertical‐resolution aerosol profiles from measurements of the scattered solar radiation in the 290–1000 nm spectral range. The OMPS LP instrument observes the atmosphere through three slits (separated by 250 km at the tangent point), providing global daily coverage (including the tropics) (Jaross et al., 2014). The OMPS LP operational algorithm retrieves the aerosol extinction coefficient profiles at 510, 600, 674, 745, 869, and 997 nm wavelengths from cloud top to ∼38 km. The retrieval algorithm relies on altitude‐normalized radiances to minimize retrieval errors due to instrument calibration uncertainties, straylight contamination, and surface/cloud reflectance (Taha et al., 2021). The normalization altitude is selected at 38.5 km, which is high enough where aerosol loading is generally at a minimum but not so high that the straylight effect becomes significant. Other instruments are described in the Supporting Information S1.

In this work, we use the newly developed Version 2.1 OMPS LP aerosol retrieval algorithm, which checks for solution convergence at six altitudes above the tropopause or 15 km (whichever is larger). This updates the convergence check used in the Version 2.0 algorithm, which monitored convergence only at 20.5 km. This change improves the retrieval at short wavelengths (510–745 nm) in the presence of fresh volcanic plumes. We also added a new data field to the daily file, the aerosol to molecular extinction ratio, analogous to an aerosol mixing ratio (Vernier et al., 2011). The molecular ratio is the air density divided by the Rayleigh scattering cross‐section.

Soon after the Hunga‐Tonga eruption, it became clear that the nominal OMPS LP 38.5 km normalization altitude used in the standard V2.1 algorithm was inadequate for certain events. Anomalous profiles with very low aerosol extinction coefficient appeared in the vicinity of the island's location, caused by the presence of aerosol at or above the normalization altitude. To address this issue, we set up special processing of OMPS LP measurements that normalizes the radiances at 44.5 km altitude. While the new processing will allow retrievals up to 44 km when the aerosol is present, the quality of the aerosol profiles will be slightly affected by additional straylight, especially for 997 nm at high latitudes and high altitudes where there is very little aerosol. Because of the effect of straylight on the longer wavelengths, we recommend avoiding the 997 nm retrievals for the V2.1 special processing. In addition, users should only use this version when looking at aerosol above 36 km. The standard V2.1 released data still provides accurate aerosol retrievals when the volcanic cloud is below 36 km, which is the case for the main volcanic cloud.

## 3 Results

### 3.1 Aerosol Measurements Above 36 km

**Figure 1:**
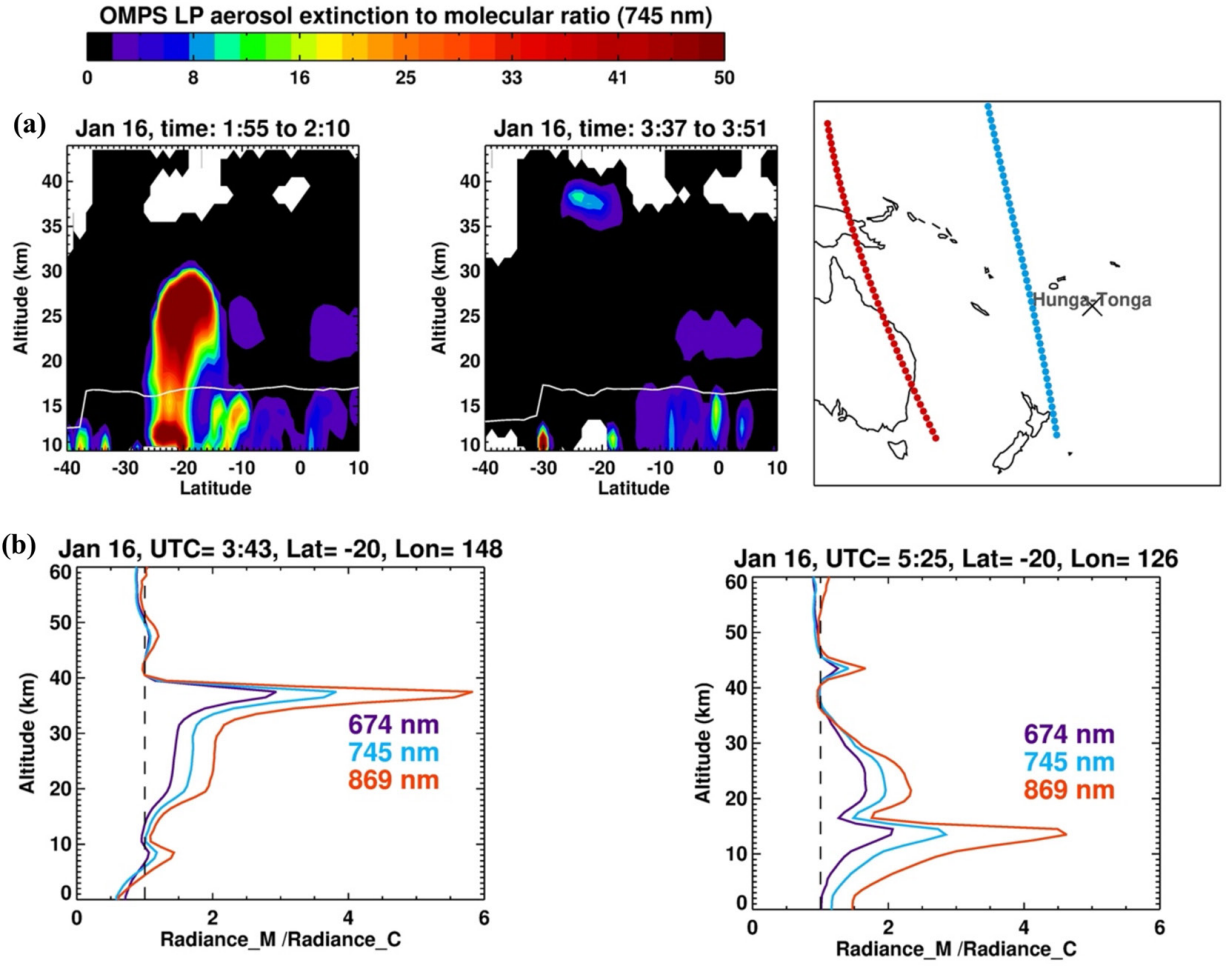
(a) Plot of Ozone Monitoring and Profiler Suite aerosol extinction to molecular ratio profiles measured on 16 January 2022, 1 day after the main volcanic eruption. The right panel is the measurement's location. Blue is for the left panel, and red is for the center. The white lines in the left and center panels are the tropopause altitudes derived using the NASA Global Modeling and Assimilation Office near‐real time atmospheric analyses. (b) Plot of the measured to calculated radiance ratio (assuming aerosol‐free atmosphere) profile for three wavelengths measured for two profiles taken on the same day.

In addition to OMPS LP, the volcanic eruption was observed by several other Earth‐observing satellites, including GOES‐17 and Himawari‐8, which provided a continuous sampling of the initial plume every 10 min. These observations were used to estimate that the plume had reached 55–58 km altitude (Carr et al., 2022; Proud et al., 2022). Bates and Carlowicz (2022) speculated that the highest parts of the plume are expected to sublimate immediately because of the arid conditions in the mesosphere. OMPS LP missed the volcanic cloud on 15 January; however, it first observed the volcanic plume on 16 January, ∼2:00 UTC, by which time the high‐altitude part of the plume in the geostationary satellites was no longer readily visible (Carr et al., 2022). OMPS LP measurements of the aerosol extinction to molecular ratio show that the bulk of material injected from the eruption was at altitudes between 20 and 30 km (Figure 1a, left panel). A second OMPS LP orbit (Figure 1a, middle panel) confirmed that a small part of the volcanic cloud remained between 36 and 40 km altitudes. We also inspected OMPS Level 1 sun‐normalized radiance measurements to ensure that they captured all high‐altitude fragments of the plume that were otherwise missed by the OMPS LP retrieval algorithm. We found two orbits on 16 January that detected the volcanic material at altitudes at or above the new normalization altitude of 44.5 km. The first was detected above the 40 km cloud seen in Figure 1a, middle panel. Figure 1b shows the ratio of the measured to calculated radiance assuming an aerosol‐free (or pure Rayleigh) atmosphere for a single event and three wavelengths (674, 745, and 869 nm). A ratio greater than unity suggests the presence of aerosol or cloud. The figure shows a weak aerosol layer between 46 and 50 km detected by the three wavelengths. This layer was also measured by adjacent images and all three OMPS LP slits (not shown). OMPS also measured another weak layer between 42 and 46 km at longitude 126°E (Figure 1b, right panel). Both layers were at or above the new normalization altitude, which prevented the OMPS LP retrieval algorithm from producing any meaningful aerosol profile. Nonetheless, this confirms the persistence of the very‐high altitude portion of the volcanic plume on the second day after the main eruption.

On 17 January, OMPS LP also detected the volcanic cloud at 40–42 km (99°E) and 42–44 km (78°E) (Figure S1 in Supporting Information S1). By then, the layers above 45 km were either evaporated, descended to lower altitudes, or were outside the OMPS LP detection limit. January 27 was the last time OMPS LP measured the volcanic layer above 40 km. On the next day, the signal became indistinguishable from the background noise. By contrast, the space‐based CALIPSO lidar observed the volcanic cloud above ∼31 km on one occasion, on 15 January, at altitudes 35–40 km (Figure S2 in Supporting Information S1). Otherwise, it was undetectable above 31 km because of CALIPSO's low signal‐to‐noise ratio in the stratosphere (Kar et al., 2019). The SAGE III/ISS occultation measurements taken from the International Space Station observed the plume above 35 km on two different days, 17 and 19 January 2022 (Figure S3 in Supporting Information S1).

**Figure 2:**
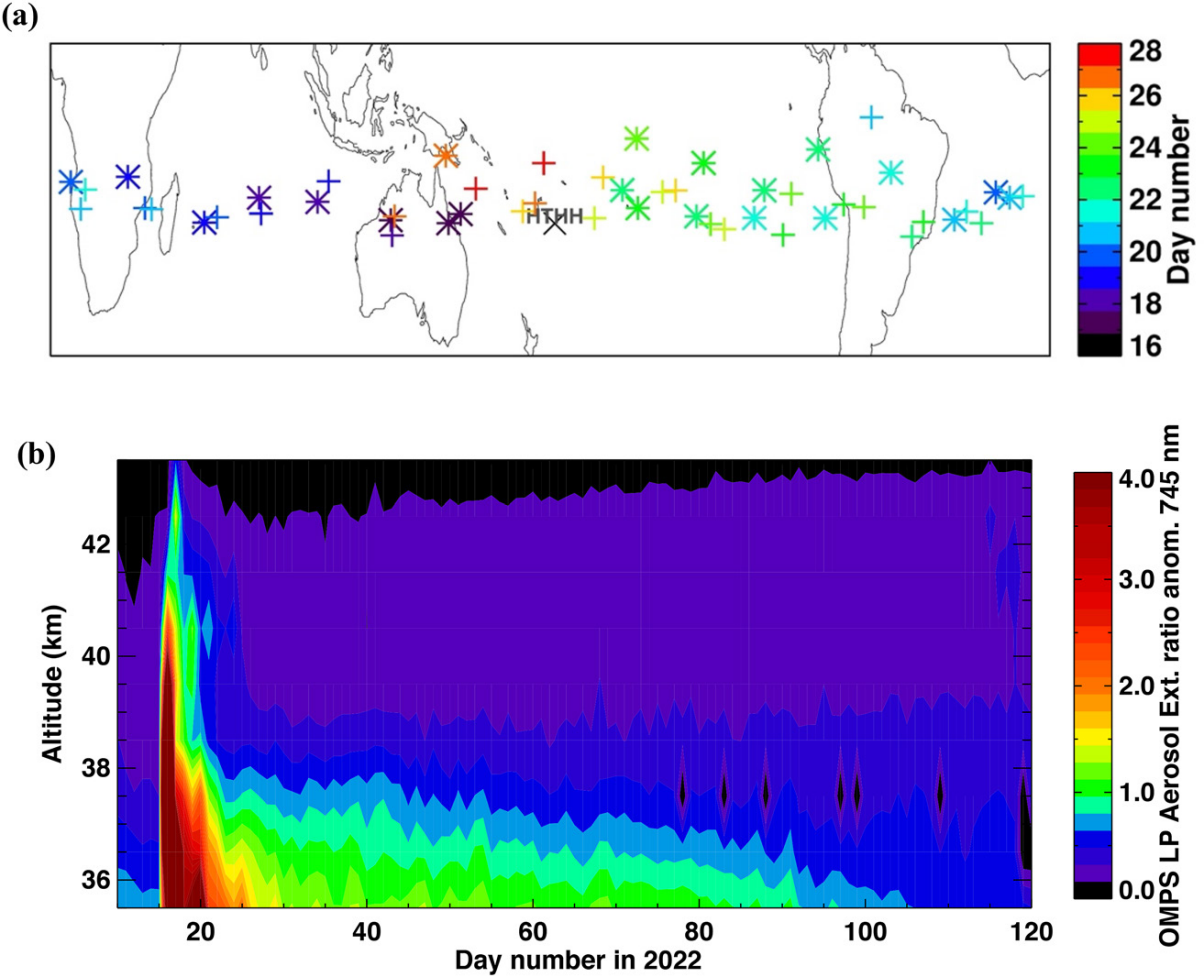
(a) Locations of Ozone Monitoring and Profiler Suite measurements with elevated aerosol values above 36.5 km during the first 2 weeks. The color scale is the day number in January. Asterisk is for layers above 40 km, and plus is for layers below 40 km. (b) Time series of the aerosol extinction to molecular ratio (745 nm) zonal mean anomalies (25°S to 0°S) for the first 4 months of 2022, where the extinction ratio at each altitude exceeds its average value by four times the standard deviation.

OMPS was able to observe the plume of enhanced aerosol above 40 km for 2 weeks following the eruption (Figure 2a). Initially, it was observed over Australia on the 16th (at 44 and 50 km), and then completely circumnavigated the globe in a week. In contrast, layers observed below 40 km took in excess of 10 days to complete a global circuit. However, they also remained measurable for 3 months. This is consistent with the vertical wind shear at these levels and with the higher altitude aerosol layer moving faster eastward than at lower altitudes (Figure S4 in Supporting Information S1). While there was some spread in latitude, particularly in a northward direction, the aerosol in these layers primarily remained in tropical latitudes throughout their observation.

We observed the volcanic plume evolution in the upper stratosphere for the first 4 months post‐eruption. Figure 2b shows the time series of the aerosol extinction to molecular ratio anomalies. The data show that aerosol levels decrease rapidly, particularly above 38 km, and return to near normal levels by day 20. On the other hand, the decrease between 35 and 38 km is slower, and the aerosol extinction remained elevated through day 100. The progression of this change suggests that some combination of sedimentation and evaporation in this ordinarily arid part of the stratosphere is slowly removing this material, thus making it undetectable (Hofmann et al., 1985; Kremser et al., 2016). While the presence of aerosol at these layers is highly unusual and interesting, the extinction levels are a tiny fraction of the main volcanic layer, and their relevance to climate and chemistry is likely low.

### 3.2 Aerosol Measurements of the Main Volcanic Layer

**Figure 3:**
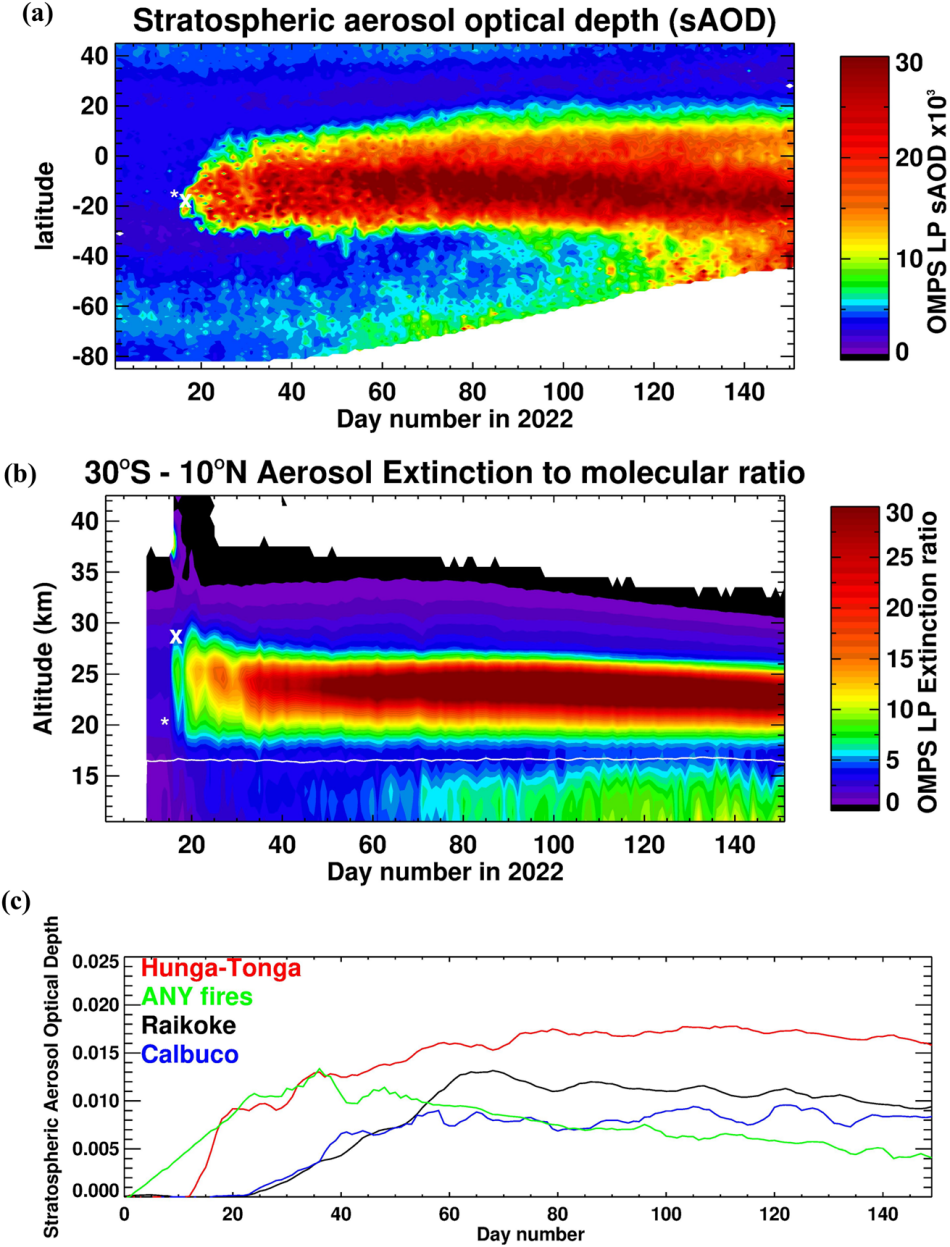
(a) Plot of Ozone Monitoring and Profiler Suite stratospheric optical depth (sAOD) x10^3^ zonal mean at 869 nm for 2022. (b) The extinction to molecular ratio zonal mean profiles between 30°S and 10°N for the same period. The white line is the tropopause altitude. The asterisk and x in both figures are for the time and location of the two eruptions on 13 and 15 January. (c) The total sAOD at 869 nm, for Hunga‐Tunga (2022) between 30°S and 15°N, Australian New Year fires (ANY) (2020) between 20°S and 90°S, Raikoke (2019) between 30°N and 90°N, and Calbuco (2015) between 20°S and 90°S, for the first 150 days, starting at the beginning of each month when the event occurred.

OMPS LP measured the first eruption on 13 January, revealing a small volcanic layer between 18 and 20 km (Figure S5 in Supporting Information S1), dwarfed by the amount of aerosol injected on the 15th. Soon after, both volcanic materials started mixing, and it became increasingly difficult to separate them. Figure 3 depicts the stratospheric aerosol optical depth (sAOD) (a) and the aerosol extinction to molecular ratio burden for the first 5 months after the eruption (b). Figure 3a shows that the bulk of the plume was transported zonally within the stratospheric zonal wind and remained trapped within the transport barrier of the tropical pipe (SPARC, 2006) due to the zonal symmetry of summer stratospheric circulation (Legras et al., 2022). Figure 3b shows that the main volcanic layer was between 20 and 26 km altitudes and exhibited a rapid descent of the aerosol layer during the first few weeks and a second slower descent in April, which were also noted by (Legras et al., 2022; Schoeberl et al., 2022). During the first few days post‐eruption, the measured sAOD loading was low because of the limited OMPS sampling of the volcanic cloud and the zonal averaging. The injection of a large amount of water from this eruption is expected to speed up the change of SO_2_ into sulfate aerosol and increase the rate of aerosol growth (LeGrande et al., 2016; Zhu et al., 2022), which might explain why the measured sAOD for this event is more than double that for the 2015 Calbuco eruption (Figure 3c) despite having a comparable amount of SO_2_ injected (Bègue et al., 2017; Solomon et al., 2016; Stone et al., 2017). The highest levels of sAOD detected by OMPS LP over the last 10 years were observed during this eruption (see Figure 3c). By this measure, it is the largest volcanic eruption since the 1991 eruption of Mt. Pinatubo (Leblanc et al., 2020).

**Figure 4:**
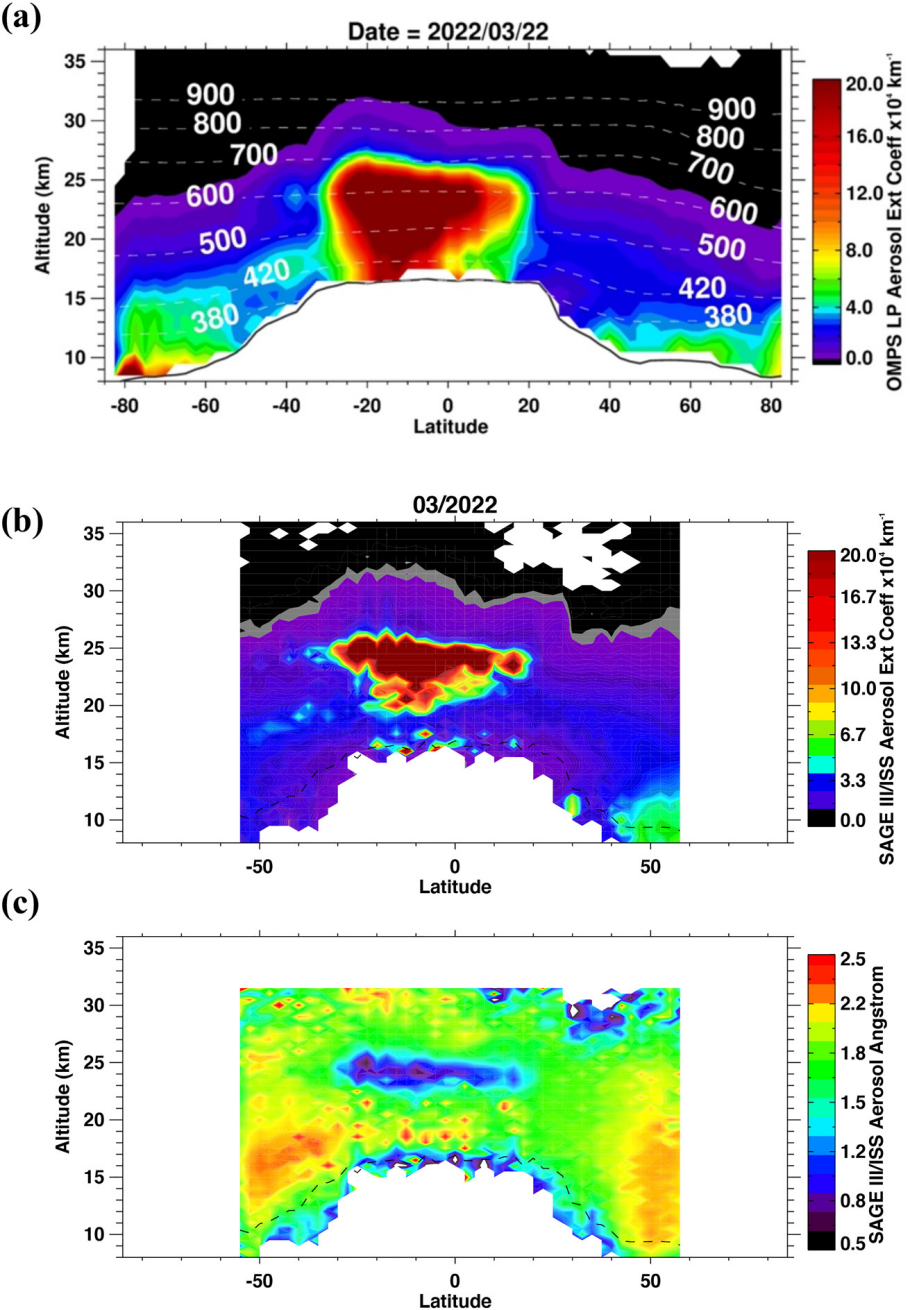
(a) Ozone Monitoring and Profiler Suite zonally averaged aerosol extinction profiles at 997 nm on 22 March 2022. The black line denotes the mean tropopause altitude, and the white contours are the mean potential temperature (K). (b) Same as (a) but for SAGE III/ISS aerosol extinction monthly zonal mean at 1022 nm for March 2022. (c) SAGE III/ISS monthly zonal mean Angstrom exponent for the same month.

Figure 3a also shows the transport of small parts of the volcanic plume toward the south. Figure 4a shows the zonally averaged aerosol extinction profiles on 22 March 2022, highlighting the poleward and downward isentropic transport of the volcanic aerosol in the SH. There is also an isentropic transport at *θ* = 600 K (or altitude ∼ 24 km), where a small aerosol layer is sheared off via an anticyclone and transported to midlatitude (see Figure S6 in Supporting Information S1). The poleward transport of the aerosol is expected to further increase during the SH winter when the Brewer‐Dobson circulation in the tropics is enhanced, which is almost certain to influence this year's ozone hole (Solomon et al., 2016; Zhu et al., 2018). By April, small parts of the volcanic plume have also reached the NH pole (see Figure S7a in Supporting Information S1). The small aerosol layer was first measured on 28 March (Figure S7c in Supporting Information S1), breaking off via an anticyclone (Figure S9a in Supporting Information S1). Figures S9a–S9c in Supporting Information S1) reveal the poleward transport of the plume after being trapped in the eastern part of the spring arctic vortex and reaching the NH pole on 4 April (Figure S8d in Supporting Information S1). Back trajectories from the aerosol layer show that it took 8 days for the layer to reach the NH pole once it was separated from the main volcanic plume (Figures S7 and S8 in Supporting Information S1).

### 3.3 The Volcanic Aerosol Properties

We use SAGE III/ISS multispectral measurements to characterize the Hunga‐Tonga volcanic plume. SAGE III/ISS is known for its highly accurate aerosol extinction retrieval and is widely used to infer information of the aerosol particle properties (Das et al., 2021; Kloss et al., 2021; Thomason et al., 2021). Figure 4b is the monthly zonal mean of the SAGE III/ISS aerosol extinction measured in March 2022 at 1022 nm. Despite its sparse sampling, March 2022 was the first month SAGE III/ISS managed to map the volcanic cloud when fully dispersed in the tropics. Although the figure is averaged for an entire month, it shows reasonable agreement with OMPS LP profiles shown in Figure 4a, with differences most likely caused by the monthly zonal averaging, instruments' sampling, and vertical resolution differences, which are ∼0.7 km for SAGE III/ISS compared to ∼1.6 km for OMPS LP. Similar to OMPS LP, SAGE III/ISS shows that the main volcanic plume is in the tropics between 19 and 25 km, with small parts transported to the SH. Figure 4c is similar to 4b but for the Angstrom exponent (AE), calculated using the extinction coefficient at 520 and 1022 nm. The figure shows an AE between 0.5 and 1.0 for the main aerosol layer 23–25 km, indicating the presence of large particle size compared to the surrounding stratospheric aerosol layer. We estimate the aerosol particles median radius of 0.21–0.28 μm, assuming sulfuric acid particles and bimodal lognormal size distribution (Figure S10b in Supporting Information S1). However, it also shows a larger AE of 2–2.4 below the peak of the aerosol layer between 19–22 km and for the aerosol that is transported to the SH, denoting small particles outside the peak of the aerosol layer (Figure S11 in Supporting Information S1), with particles median radius of 0.08–0.1 μm, assuming unimodal lognormal distribution (Figure S10a in Supporting Information S1). CALIPSO depolarization ratio for the main aerosol layer shows very low values of less than 0.1, confirming that the measured layer is mainly composed of spherical sulfate particles (Figure S12 in Supporting Information S1).

Furthermore, TROPOMI UV instrument measurements (Veefkind et al., 2012) indicate that the volcanic cloud is mainly composed of SO_2_ with very little ash (see Figure S13 in Supporting Information S1), which was also supported by (Sellitto et al., 2022) using Himawari images. The larger particle size and low depolarization ratio of the aerosol layer are possibly due to coagulation of the sulfate aerosol particulate and condensation on pre‐existing aerosols (LeGrande et al., 2016; Thomason et al., 2021). Kloss et al. (2022) used in situ balloon measurements to show that the aerosol particle size within the main plume was between 0.5 and 1 micron and composed primarily of sulfate aerosol with a small component of absorbing aerosol.

The larger Angstrom coefficient shown in Figure 4c suggests smaller particle sizes at lower altitudes and in the SH, probably caused by the separation of parts of the SO_2_ from the water‐rich main plume at higher altitudes, which resulted in the formation of the smaller sulfate particles at these altitudes.

## 4 Summary and Conclusions

The Hunga Tonga‐Hunga Ha'apai eruption reached a record‐breaking altitude that had never been seen before in the satellite era. The plume altitude was the highest ever measured by OMPS LP at 50 km, and the sAOD is the largest measured over the past 10 years. Because of its high sensitivity to aerosols, OMPS LP continued to track the volcanic plume above 36 km for several weeks, when most instruments lost the ability to detect it. The uppermost parts of the plume above 40 km circulated the globe in 7 days and remained visible for 12 days, while the aerosol levels above 36 km remained elevated for 90 days. OMPS LP's unique measurements of the high‐altitude parts of the volcanic plume will enable the scientific community to understand its short‐ and long‐term impact on the atmosphere.

OMPS LP continued to provide 3‐D global measurements of the volcanic plume and account for the horizontal and vertical distribution of aerosols in the stratosphere. Although this eruption emitted a similar amount of SO_2_ (∼0.4 Tg) to Calbuco, it produced nearly twice as much aerosol likely because of the large amount of water injected from this submarine eruption. OMPS LP aerosol measurements will enable the global climate models to accurately predict the atmospheric and climate impact of the eruption and its effect on the ozone layer. It is expected that significant parts of the aerosol layer will make their way to the SH pole this winter and play an important role in influencing the 2022 ozone depletion.

## Supporting information

Supporting Information S1

## Data Availability

OMPS‐LP V2.1 data is available from https://disc.gsfc.nasa.gov/datasets/OMPS_NPP_LP_L2_O3_DAILY_2/summary. OMPS‐LP high altitude special processing is available at https://avdc.gsfc.nasa.gov/pub/data/satellite/Suomi_NPP/L2/LP-L2-AER-45km/. CALIPSO data files are available at https://asdc.larc.nasa.gov/project/CALIPSO. TROPOMI level 2 product maps are available at https://sacs.aeronomie.be/. SAGE III/ISS data is available from https://asdc.larc.nasa.gov/project/SAGE%20III-ISS.
