# Supplementary material for: Tracking the 2022 Hunga Tonga‐Hunga Ha'apai Aerosol Cloud in the Upper and Middle Stratosphere Using Space‐Based Observations
Source: Geophys Res Lett. 2022 Oct 4;49(19):e2022GL100091. doi: 10.1029/2022GL100091 (PMC9786872; doi:10.1029/2022GL100091)
Supplement: Supplementary file 1 — Supporting Information S1 [file GRL-49-e2022GL100091-s001.pdf]

Geophysical Research Letters

Supporting Information for

Tracking the 2022 Hunga Tonga-Hunga Ha'apai aerosol cloud in the upper and middle stratosphere using space-based observations

G. Taha[1,2], R. Loughman[3], P. R. Colarco[2], T. Zhu[4], L. W. Thomason[5], and G. Jaross[2]

[1]Morgan State University, Baltimore, MD, USA

[2]NASA Goddard Space Flight Center, Greenbelt, MD, USA

[3]Hampton University, Hampton, USA

[4]Science Systems and Applications, Inc., Lanham, MD, USA

[5]NASA Langley Research Center, Hampton, VA, USA]

Contents of this file

Text S1
Figures S1 to S12

S1 Instruments and data Descriptions:

SAGE III/ISS

The Stratospheric Aerosol and Gas Experiment (SAGE) III is a solar occultation instrument onboard the international space station (ISS) (Thomason and Taha, 2003). It measures high-resolution vertical profiles of multiple gaseous species as well as the aerosol extinction at various wavelengths. It provides about 15 sunrise and 15 sunset events per day, mostly between 60°S and 60°N. In this study, we used V5.2 data files.

CALIPSO

The Cloud-Aerosol Lidar and Infrared Pathfinder Satellite Observation (CALIPSO) is a spaceborne backscatter lidar instrument (Winker et al., 2010). It provides global measurements of vertically resolved aerosol- and cloud-attenuated backscatter coefficients at 532 and 1064 nm. It also provides depolarization ratio, which can give information on the particle type. In this study, we used level 1 V3.41 data files

TROPOMI

The TROPOspheric Monitoring Instrument (TROPOMI) is a nadir viewing imaging spectrometer that measures the Earth's backscattered radiance between the ultraviolet and short infrared range (Veefkind et al., 2012). It provides high-resolution daily global maps of SO_2 and absorbing aerosol index (AAI), among other gaseous species. In this study, we used BIRA SO2 and AAI near-real-time images.

S2 Methods

Mie calculation

Aerosol particle size calculations shown in Figure S10 were derived using Mie calculations, assuming spherical particles composed of a mixture of sulfuric acid (75%) and water (25%) (Kremser et al., 2016), using the refractive index of 1.4542,1.4473 for 525 and 1020 nm respectively, for either unimodal or bimodal particle size distribution. We varied the median radius between r_m = 0.08 - 0.27 (μm) for the unimodal log-normal particle distribution. As for the bimodal distribution, we used a fine mode radius of 0.08 μm and varied the coarse mode between r_m = 0.08 - 0.55 (μm). The distribution width was 1.6 for all cases. The theoretical values shown in Figure S10 were used to derive the SAGE III/ISS particle size.

OMPS LP daily maps

OMPS LP daily maps were constructed by gridding OMPS measurements to a uniform grid of 1.5^o Latitude x 24° longitude and interpolated to potential temperature levels. Wind Streamlines are derived using the GMAO GEOS-5 forward processing data and interpolated to potential temperature levels.

Back trajectories are calculated using the NOAA Air Resources Laboratory (ARL) for the provision of the HYSPLIT transport and dispersion model READY website (https://www.ready.noaa.gov)

Figures

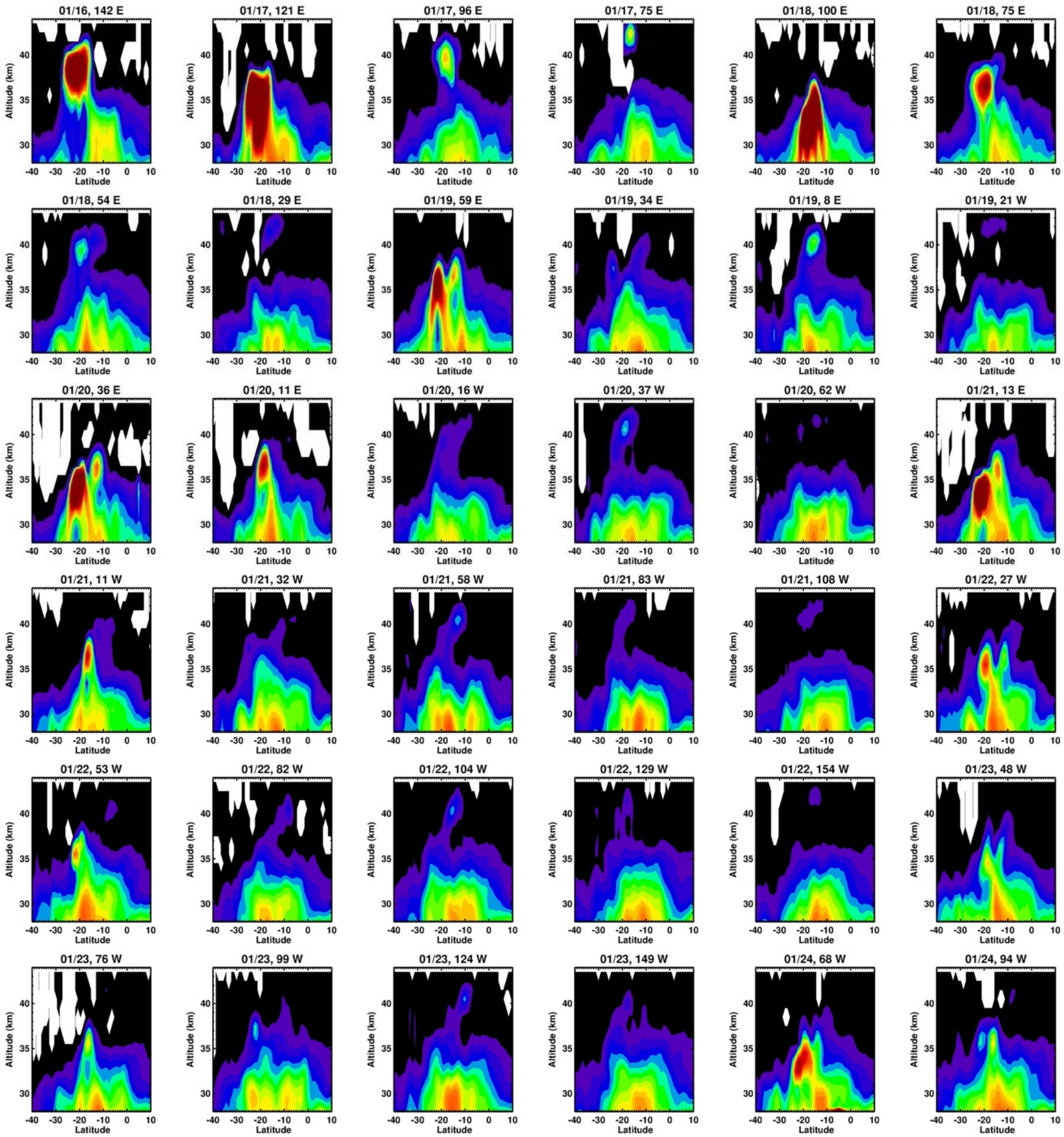
01/16, 142 E
01/17, 121 E
01/17, 96 E
01/17, 75 E
01/18, 100 E
01/18, 75 E
01/18, 54 E
01/18, 29 E
01/19, 59 E
01/19, 34 E
01/19, 8 E
01/19, 21 W
01/20, 36 E
01/20, 11 E
01/20, 16 W
01/20, 37 W
01/20, 62 W
01/21, 13 E
01/21, 11 W
01/21, 32 W
01/21, 58 W
01/21, 83 W
01/21, 108 W
01/22, 27 W
01/22, 53 W
01/22, 82 W
01/22, 104 W
01/22, 129 W
01/22, 154 W
01/23, 48 W
01/23, 76 W
01/23, 99 W
01/23, 124 W
01/23, 149 W
01/24, 68 W
01/24, 94 W
Altitude (km)
Latitude

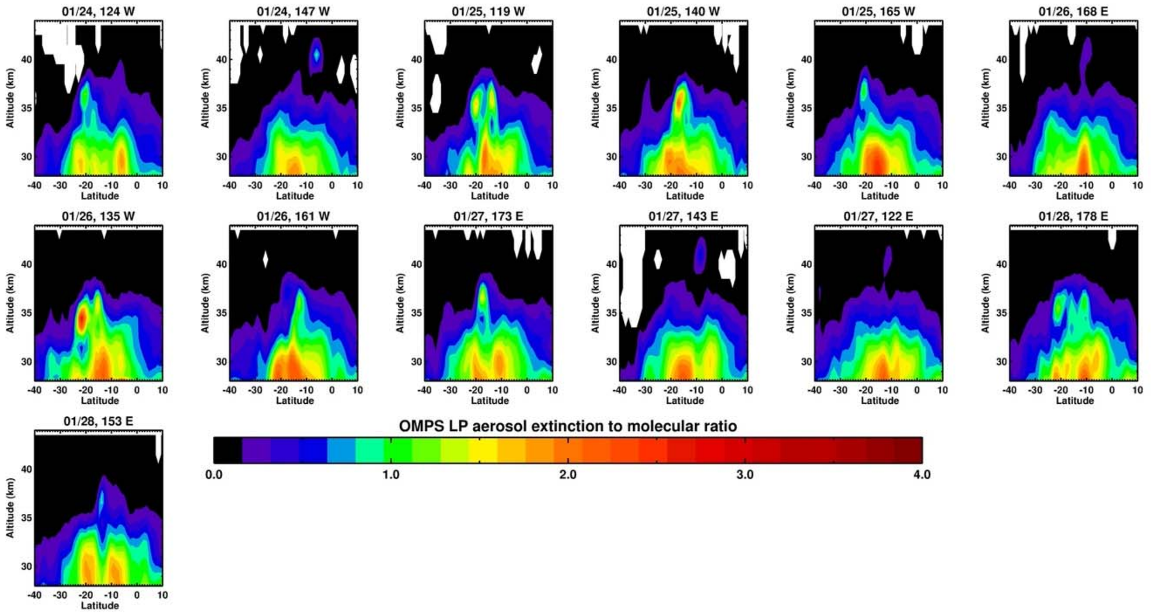

Figure S1. Plots of selected OMPS LP aerosol extinction to molecular ratio profiles measured during January 2022, which detected the uppermost part of the volcanic cloud. The longitude in the header of each panel corresponds to the equator crossing point.

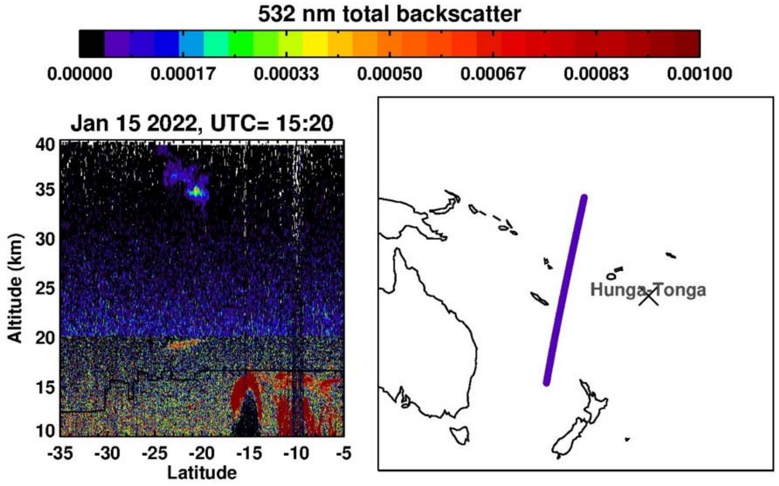

Figure S2. CALIPSO backscatter measurement on Jan 15, which detected the volcanic cloud between 35 and 40 km near ~ 20º S latitude. The right panel is the measurement's location.

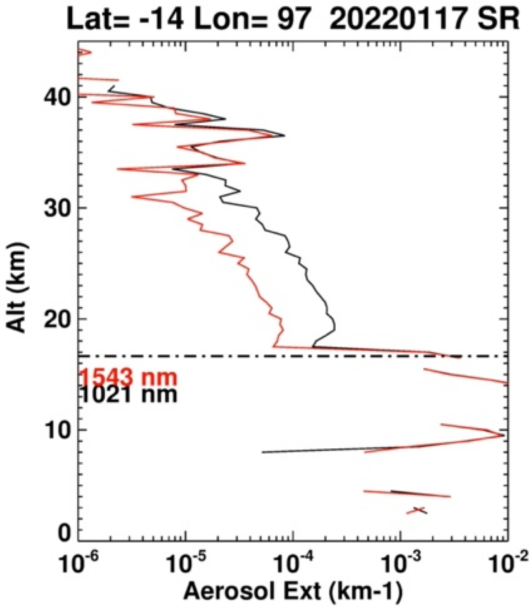

Figure S3. SAGE III/ISS aerosol profile measured on Jan 17, 2022, for two wavelengths, 1022 and 1543 nm. The dashed line is the tropopause altitude.

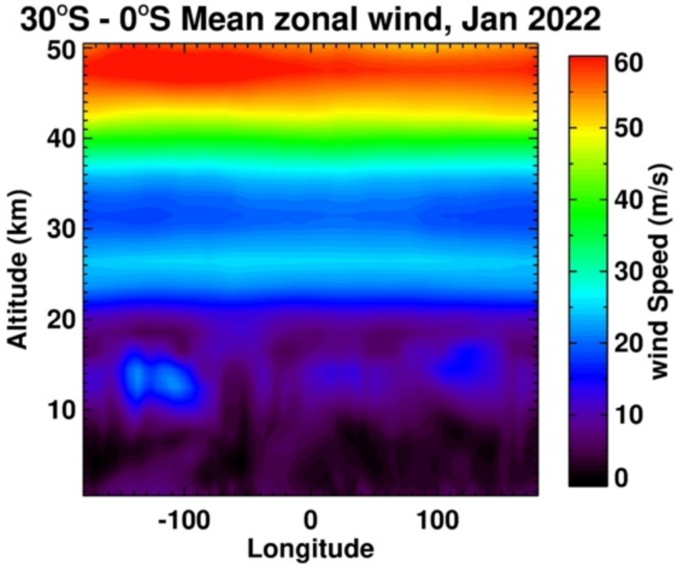

Figure S4. Plot of MERRA-2 mean zonal wind speed profiles between 30°S and 0°S for January 2022.

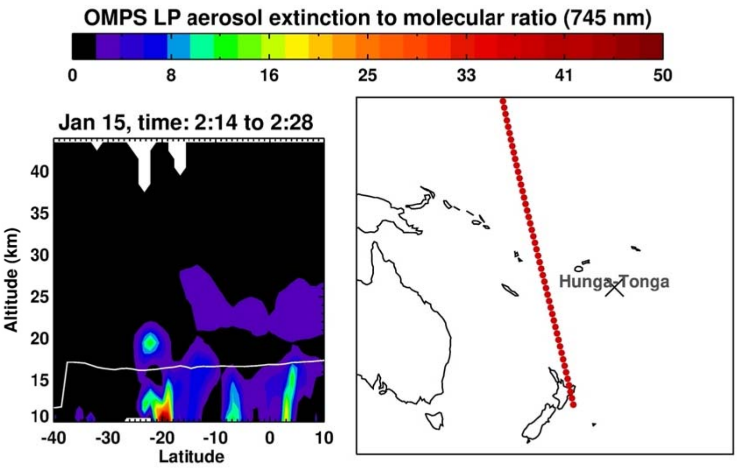

Figure S5: Plot of OMPS LP aerosol extinction to molecular ratio profiles measured on 15 January 2022. The right panel is the measurement's location. The white line in the left panel is the tropopause altitude.

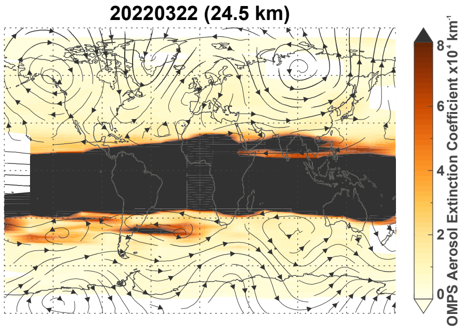

Figure S6. OMPS LP aerosol extinction at 24.5 km (997 nm) for March 22, 2022, superimposed is GMAO wind streamlines.

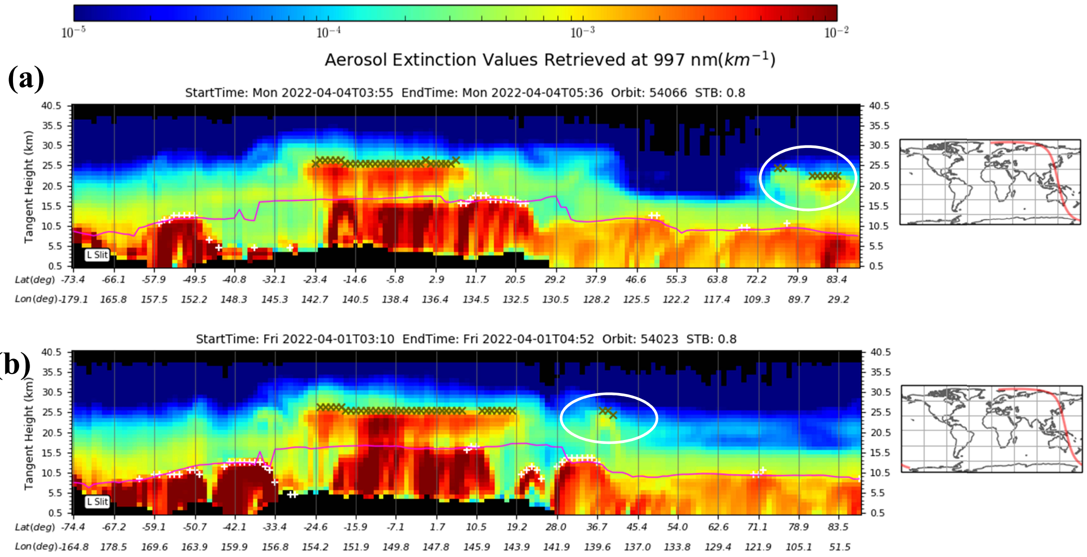

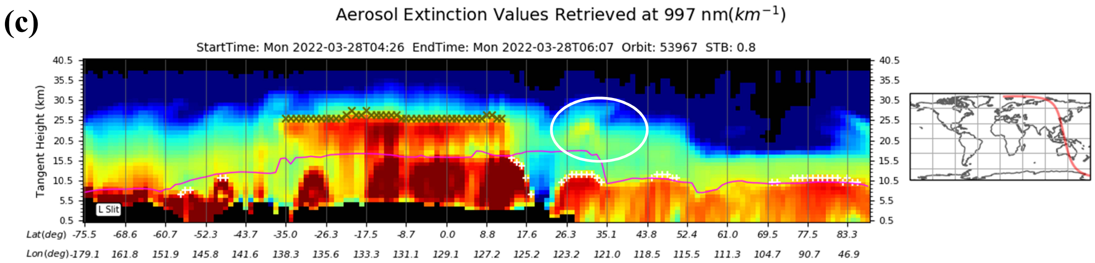

Figure S7. OMPS LP aerosol extinction individual orbital plots for April 4 (a), 1 (b), and March 28 (c), 2022, at 997 nm. The purple line is the tropopause altitude, white pluses are clouds, and green crosses are enhanced aerosol layers. OMPS orbital tracks are shown to the right. White circles highlight the aerosol layer that broke off the main plume and ended up over the NH pole.

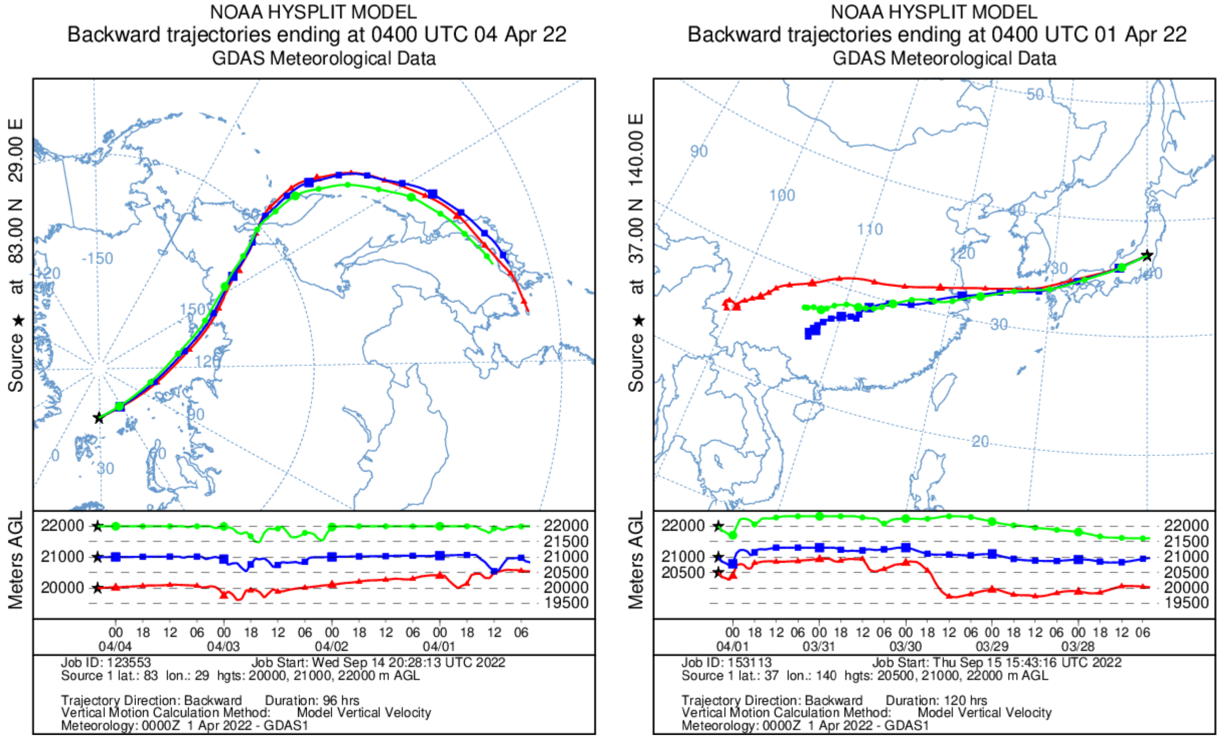

Figure S8. The left panel is a plot of the backward trajectory initiated on April 4, 2022, at the location and altitude of the aerosol layer detected over the NH pole (Figure S7a). The trajectory parcels landed on April 1 over Japan. The right panel is for the back trajectory initiated on April 1, 2022, at the location and altitude of the aerosol layer shown in (Figure S7b) and where the previous trajectories ended. The second trajectory parcels landed over China on March 28, 2022, in the tropics and at the location of the aerosol layer shown in Figure S7c, thus confirming that the Honga-Tonga volcanic cloud is the origin of the aerosol layer measured over the NH Pole on April 4.

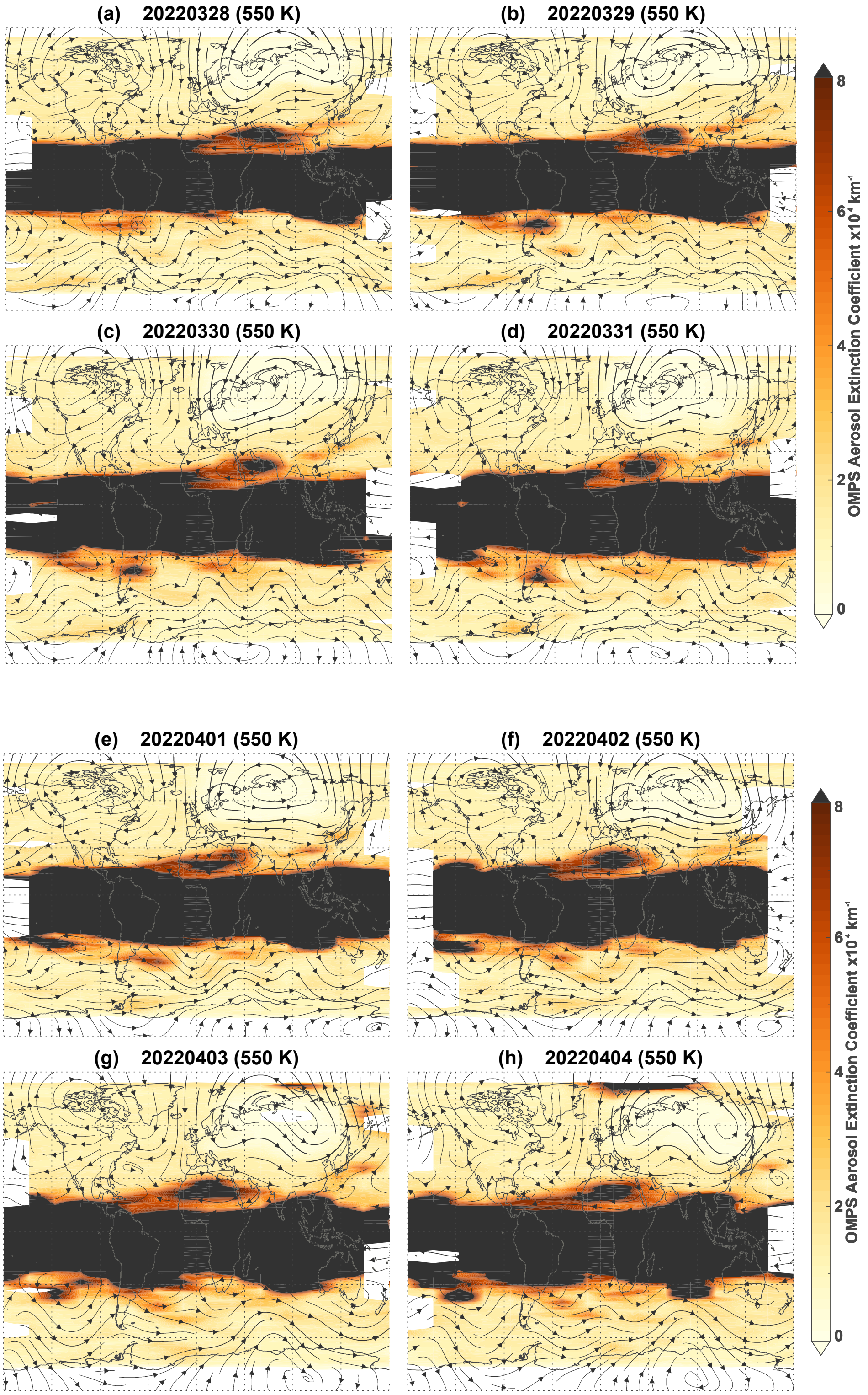

Figure S9. OMPS LP aerosol extinction (997 nm) at 550 K levels (~21 km) for March 28 to April 4, 2022, superimposed is GMAO wind streamlines.

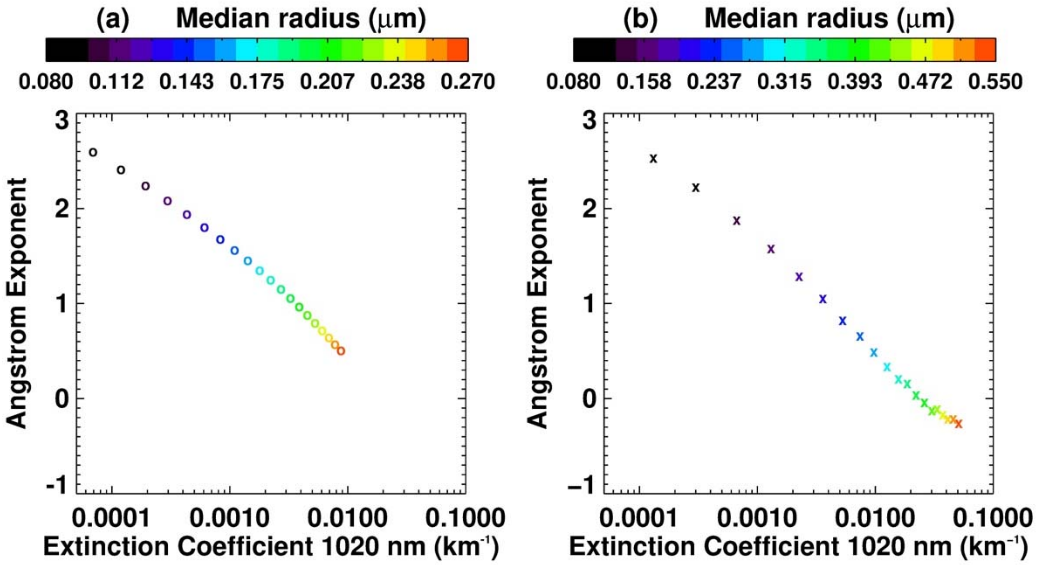

Figure S10. Angstrom exponent vs. aerosol extinction coefficient at 1020 nm simulated for sulfate aerosol colored by the particle's median radius (r_m) values. Plot (a) is for single mode log-normal particle distribution, with a median radius between r_m = 0.08 - 0.27 (μm). Plot (b) is for bimodal log-normal particle distribution between r_m = 0.08 - 0.55 (μm). The distribution width was 1.6 for all cases.

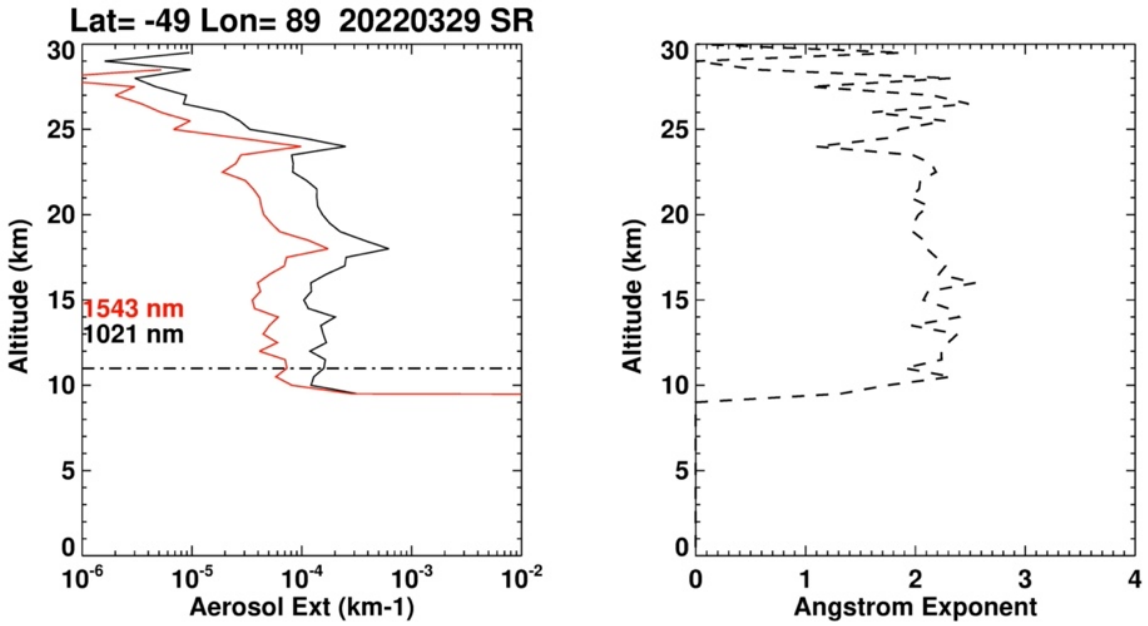

Figure S11. SAGE III/ISS aerosol profile measured on March 29, 2022, for two wavelengths, 1022 and 1543 nm (left panel). The dashed line is the tropopause altitude. The right panel is the angstrom exponent. The figure shows two volcanic aerosol layers at

24 and 17 km. The 24 km layer appears to be composed of larger particles, while the 17 km is mostly of smaller particles.

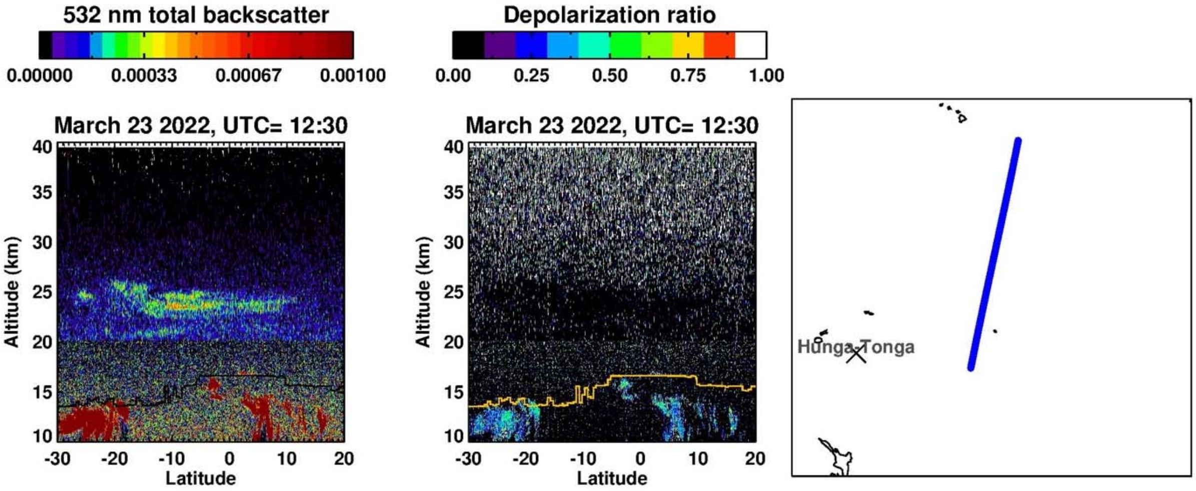

Figure S12. CALIPSO backscatter measurement on March 23, 2022, which detected the volcanic cloud between 20 and 26 km (left). A very low (<0.1) depolarization ratio (middle) was detected for the volcanic cloud. The right panel is the measurement's location.

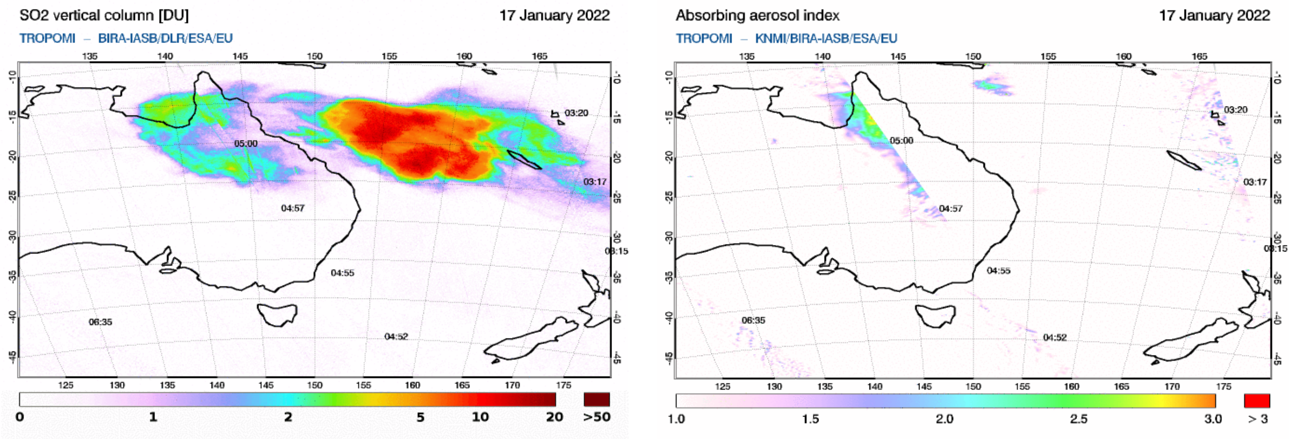

Figure S13. TROPOMI UV SO_2 (left) and Aerosol Index (right) observed for January 17, 2022. Both figures show that the Hunga-Tonga eruption was rich in SO_2 and poor in ash. High absorbing aerosol index (AAI) points to UV absorbing aerosols such as smoke or ash (Krotkov et al., 1997). The SO_2 cloud disappeared by 22 January while the AAI remained near zero for the same period.
